# Emergency specialists’ perceptions of climate-related health and education in South Africa

**DOI:** 10.4102/jcmsa.v3i1.214

**Published:** 2025-09-16

**Authors:** Elzarie Theron, Gina Ziervogel, Willem Stassen

**Affiliations:** 1 Division of Emergency Medicine, Faculty of Health Sciences, University of Cape Town, Cape Town, South Africa; 2 Department of Environmental and Geographical Science, Faculty of Science, University of Cape Town, Cape Town, South Africa; 3 African Climate and Development Initiative, Faculty of Science, University of Cape Town, Cape Town, South Africa

**Keywords:** climate change, health education, climate-related medical curriculum, emergency care preparedness, climate change adaptation, South Africa

## Abstract

**Background:**

Climate change exacerbates existing social inequalities, placing a disproportionate burden on already vulnerable populations. As emergency care often functions as the initial point of contact for affected patients, it is crucial that these specialists understand the impacts and are appropriately trained to respond. However, it is unclear whether specialists in South Africa are equipped with the knowledge and preparedness to do so in clinical practice.

**Methods:**

This study followed a quantitative survey design to describe emergency medicine registrars’ and physicians’ perceptions about climate change and health, climate-related health education, and self-perceived preparedness to respond to these impacts.

**Results:**

The majority (80%, *n* = 16) of 20 participants (combining ‘probably’ and ‘definitely’) recognised climate-related health impacts; 70% (*n* = 14) believed that it would impact their own health, and 95% (*n* = 19) felt that it would impact their patients’ health (combining ‘agree’ and ‘strongly agree’). However, none of the participants had received any formal training on the topic, and only 3 participants (15%) indicated that they ‘probably’ have enough information to prepare and respond to the health impacts of climate change. When asked about key priorities to improve preparedness, 65% (*n* = 13) highlighted the importance of education and information.

**Conclusion:**

The findings suggest a gap in preparedness and underscore the need for a climate-responsive emergency care education.

**Contribution:**

This is the first study in South Africa to describe emergency medicine specialists’ perceptions and preparedness in relation to climate change and health, establishing a foundation for future curriculum development.

## Introduction

Climate change has several direct and indirect impacts on health^1^ that occur on top of existing social inequalities,^2^ placing a disproportionate burden on those who are already vulnerable.^3^ This disproportionate burden raises critical climate justice concerns,^4^ particularly in countries such as South Africa, where extreme inequality persists.^5^ Recognising that emergency care often functions as the safety net for health care and the initial point of contact for patients whose health has been adversely impacted by climate change,^6^ emergency medicine specialists must understand these impacts and be appropriately educated and prepared to effectively respond to them in practice. However, it is anticipated that many emergency medicine specialists in South Africa may not have a sufficient understanding of the health impacts of climate change. This makes it essential to determine their current understanding and preparedness to address these impacts in clinical practice, so that appropriate training and education can be developed.

Few studies on the African continent have explored emergency care preparedness as it relates to the health impacts of climate change.^7^ As a result, not much is known about emergency medicine specialists’ understanding, perceptions, education needs and level of preparedness concerning climate change and health. While research exists that explores health care professionals’ (HCPs) perceptions about the link between climate change and health,^8,9,10,11,12^ it should be noted that these studies do not explicitly involve emergency medicine specialists, nor is it clear whether this group is represented in these studies. However, international research has shown that the integration of climate change into medical curricula is largely lacking.^11,13,14^ This suggests the possibility that emergency medicine specialists, much like other HCPs, may not be receiving formal education to help them prepare and respond to climate-related health impacts, and as a result, they may be insufficiently prepared to do so.

According to the Pan American Health Organisation (PAHO), there is a critical need for health care workers (HCWs) to be able to recognise and effectively treat the medical conditions attributable to climate change.^15^ Furthermore, PAHO argues that it is HCWs’ role to adapt current procedures to enable them to respond more effectively to climate-related health impacts, identify the available information on early warning systems of climate-related events and disease outbreaks, identify vulnerable groups in the population and provide appropriate risk reduction advice to patients and community members.^15^ To improve HCWs’ understanding and stay informed about the relationship between climate change and health, PAHO suggests that HCWs need to continue their training and education on the topic.^15^ Lemery et al. suggest that HCWs have three primary roles to support efforts that address climate risks.^16^ Firstly, HCWs have to protect individual and community health against the direct and indirect climate-related health impacts. Secondly, HCWs should build health care and health systems that are resilient to the threats posed by climate change by focusing on both climate change mitigation and adaptation strategies. And thirdly, they need to use their voices and scientific expertise to promote and support intersectoral solutions to the climate crisis.^16^

Integrating climate-related health education into medical curricula could enhance emergency medicine specialists’ recognition of climate-sensitive presentations, understanding of health risks and patterns exacerbated by climate change and awareness of the intersection between climate change and social determinants of health. An improved understanding of the topic could also stimulate greater engagement in the implementation of climate change mitigative and adaptive strategies in the health care system^17^ and inspire a group of trusted and influential voices in the community to advocate for climate change.^18^ This advocacy role is particularly important in addressing climate justice concerns, as emergency medicine specialists are often the first point of care for those most affected by climate-related health inequities.

### Research aim

This study aimed to describe emergency medicine registrars’ and physicians’ perceptions related to climate change and health, climate-related health education and training, and self-perceived preparedness and priorities to respond to these impacts.

## Research methods and design

### Study design

This study followed a cross-sectional descriptive survey design.

### Setting

Emergency medicine is a relatively new speciality in South Africa and was first registered in 2003.^19^ The Division of Emergency Medicine at the University of Cape Town was the first to offer the Master of Medicine in Emergency Medicine (MMed) programme,^19^ which is now being offered at six universities across the country. Emergency medicine specialist training in South Africa includes 4 years of 3-month blocks consisting of emergency medical services, intensive care, obstetrics and gynaecology, internal medicine, surgery and paediatrics.^19^ The balance of the time is spent in emergency departments (EDs).

### Study population and sampling strategy

Emergency medicine specialists work in EDs to provide treatment to patients who need immediate care. The sampling frame included all emergency medicine registrars and physicians who, at the time of the study, were registered with the Health Professions Council of South Africa (HPCSA) either as an emergency medicine registrar or as an emergency medicine physician. While the exact population size of emergency medicine registrars and physicians in South Africa at the time of data collection could not be confirmed, anecdotal reports suggested that there were approximately 200 emergency medicine registrars and physicians registered with the HPCSA at the time.

A convenience sample was drawn from a population of emergency medicine registrars and physicians studying or working in South Africa. An invitation to participate in the study was distributed using email and X (formerly Twitter). The following special interest groups were contacted via email to distribute the invitation to their members: Emergency Medicine Society of South Africa (EMSSA) and Emergency Care Society of South Africa (ECSSA). Recipients of the invitation were asked to share it with other prospective participants, who were then able to access the survey online by following the link that was included in the invitation. Information about the study, how data will be managed, the right to withdraw and voluntary participation was shared on the landing page of the online survey. Prospective participants were informed that by clicking the ‘Agree’ button at the bottom of the page, they agree that they have read the information on that page and they consent to take part in the study.

The required sample size of a study is often based on the statistical analyses that will be used. As a general rule of thumb, for descriptive analysis, using means and frequencies to describe the data, nearly any sample size is accepted as sufficient.^20^ According to Gay and Diehl (as cited in Hill^21^), for descriptive research, a sample size of 10% to 20% of the population is adequate. Therefore, based on this recommendation and the population size of 200 registrars and physicians, the intended sample size of this study was between 20 and 40 participants.

### Data collection

Data were collected using a self-administered online questionnaire (Online Appendix 1), which was available on Google Forms (Google Inc., California, United States [US]) from March 2023 to May 2023. The 26-item questionnaire consisted of a combination of Likert-type, multiple-choice, binary and free-text questions, of which 12 questionnaire items (Questions 10, 11, 12.A, 13, 14, 16, 17, 18, 19, 20, 22 and 23) were adapted from two existing surveys to align with the emergency health care focus of the study and the study population.^8,12^ For example, the term ‘physician’ was replaced with ‘emergency care providers’ (Question 11), and answer options were added for Question 12.A. In addition to the 12 adapted questions, five new questions were developed for this study to expand on existing constructs (Questions 12.B, 15.A, 15.B and 21) and to explore participants’ views on the main priorities for improving preparedness – an area of interest to the research team (Question 24). Finally, nine biographical questions were developed to capture the demographic and professional information about the study sample.

The questionnaire was pilot-tested by three health care experts prior to data collection to determine content validity. The content validity of a questionnaire refers to how representative the questions contained in the test are of the phenomenon or topic being studied.^22^ Determining content validity follows a formal assessment by subject experts to determine the appropriateness and completeness of the content and to highlight possible misunderstandings.^23^ Two of the experts are emergency medicine specialists, and the third expert specialises in climate-related health education in health sciences curricula. All three experts are practising in South Africa.

The questionnaire was in English, which is the primary medium of instruction in medical school curricula in South Africa.

The questionnaire was divided into four parts, collecting: (1) participants’ biographical information, (2) participants’ perceptions of climate change as a potential threat to health, (3) participants’ perceptions about climate change as part of medical education and training and (4) participants’ self-perceived preparedness to respond to the health impacts of climate change. The first part of the questionnaire collected biographical information of the participants. This section consisted of questions 1–9. Demographic data that were collected included medical school attended, graduation date from medical school, years active as a medical professional, graduation date or expected graduation date as an emergency physician, province currently working in, date of birth and gender.

A short definition of anthropogenic climate change and its associated impacts on the natural environment was then shown to the participants before they could continue to the second part of the questionnaire.

The second part of the questionnaire collected data about participants’ perceptions of climate change as a potential threat to health. This section included seven questions that consisted of Likert-type, multiple-choice and open-ended questions. Questions such as ‘Do you think there is a link between climate change and health?’ and ‘In what way do you think these climate change variations could impact health?’ measured participants’ perceptions of the health impact of climate change on both their and their patients’ health in the future.

The third part of the questionnaire collected data about participants’ perceptions of climate change as part of medical education and training. This section included six or seven questions, depending on participants’ answers, that consisted of Likert-type, binary, multiple-choice and open-ended questions. Questions such as ‘Did you receive formal training surrounding the health impacts of climate change during medical school?’ and ‘Do you think that you received enough formal training surrounding the health impacts of climate change?’ collected data about participants’ engagement and expectations as it relates to climate change curriculum in medical education and training.

The final part of the questionnaire collected data about participants’ self-perceived preparedness to respond to the health impacts of climate change. This section included six questions that included Likert-type, multiple-choice and open-ended questions. Questions such as: ‘If a patient asked you, How can climate change impact my health? how prepared would you feel to engage in a conversation with them?’; and ‘In your opinion, what are the main priorities to focus on that will increase preparedness to respond to the health impacts of climate change?’ collected data about participants’ self-perceived preparedness to respond to the health impacts of climate change.

This survey collected information about participants’ perceptions and opinions. This could present a threat to the validity of the study if participants responded to questions in a manner that is perceived as socially acceptable. It can, however, be expected that participants would respond truthfully about their perceptions and opinions provided that no strong social desirability or sensitivity exists about the topic when the researcher is not directly involved with data capturing (i.e., self-administered questionnaire), and the questionnaire does not collect identifiable information.^24^ In which case it can be assumed that in this study, the participants would have likely responded with honest answers to the survey questions.

### Data analysis

The data were extracted from Google Forms (Google Inc., California, US) into Microsoft Excel (Microsoft Corporation, Washington, US) for data cleaning and analysis. There were no missing data points, and as such, no entries were removed. Each participant was assigned an identification number. The quantitative data set (excluding free response answers) was imported into the Statistical Package for the Social Sciences (SPSS, IBM®, Version 28, New York, US) for analysis.

Continuous data, including year of birth, year graduated from medical school, year of expected or actual graduation as an emergency physician, years working in the health sector and years working in the emergency department, were summarised using median and interquartile range (IQR) statistics. Categorical data, including gender, medical schools attended, province currently employed in and data produced from Likert-type questions, were summarised using frequencies and percentages. Multiple-choice responses were counted and ranked, where applicable, and reported in terms of frequencies and percentages. Responses to the binary question were counted and reported using frequency and percentage. Free-text responses were analysed inductively following Elo and Kyngäs’^25^ recommendations on how to conduct quantitative content analysis. The results were compiled to address the research objectives.

### Ethical considerations

Ethical clearance to conduct this study was obtained from the University of Cape Town, Faculty of Health Sciences, Human Research Ethics Committee on 10 February 2023 (HREC REF: 065/2023).

## Results

### Study participants

Twenty participants completed the questionnaire of which two-thirds (65%, *n* = 13) identified as women and seven (35%) identified as men. The participants’ year of birth ranged from 1967 to 1993 (Median = 1989, interquartile range [IQR] = 3). Participants reported graduating from undergraduate medical school between 1993 and 2018 (Median = 2013, IQR = 4), and five (25%) participants reported having graduated as emergency physicians before 2023. Participants’ years of experience working in the health sector ranged from 5 years to 29 years (Median = 10.00, IQR = 2), and participants’ years of experience working in the ED ranged from 2 years to 26 years (Median = 7.50, IQR = 4). The majority of participants reported that they are currently employed in the Western Cape province (WC) (75%, *n* = 15), four participants (20%) are employed in Port Elizabeth (PE) and one participant (5%) is employed in Gauteng province (GP). Information about the medical schools that participants attended is contained in Table 1.

**TABLE 1:** Medical schools attended.

Medical School	Undergraduate medical schools attended		MMed medical schools attended	
	Frequency	%	Frequency	%
SUN	4	20	6	30
UCT	5	25	10	50
UL	1	5	-	-
UP	5	25	1	5
UFS	2	10	-	-
WITS	1	5	2	10
UN	1	5	-	-
UKZN	1	5	-	-
Grandfathered†	-	-	1	5

**Total**	**20**	**100**	**20**	**100**

SUN, Stellenbosch University; UCT, University of Cape Town; UL, University of Limpopo; UP, University of Pretoria; UFS, University of the Free State; UN, University of Nairobi; UKZN, University of KwaZulu-Natal; WITS, University of the Witwatersrand.

† , Accepted into the speciality preceding the availability of specialist training.^19,27^

### Perceptions related to climate change and health

Participants were asked to select the top three climate change impacts of concern for their province from a list of 10 options considered potential impacts in South Africa^28^ and an additional free-text option. The majority of participants indicated increased severity of drought (*n* = 9), not enough food (*n* = 9), changes in the geographic spread of vector-borne diseases (*n* = 8) and increased fire risks (*n* = 8). Participants who are currently employed in GP rated changes in the geographic spread of vector-borne disease as their top concern (*n* = 3), and those who are currently working in the WC rated rising sea levels (*n* = 7) and inadequate supply of food as their highest concerns (*n* = 7).

Participants had the opportunity to add free-text about the way they think these climate change impacts could influence health. All 20 participants responded to this question. Almost two-thirds (65%, *n* = 13) of the sample participants indicated that they expect shifting disease trends and a general increase in burden. One participant noted: ‘changing patterns of disease and overall increased burden of disease’ (Participant 11, man, 8 years working in ED). Other assumed changes included increased malnutrition because of ‘disruption of (the) food chain and food production due to weather’ (Participant 2, woman, 3 years working in ED); and increased climate change-related emergencies such as ‘floods leading to drowning incidents’ (Participant 3, woman, 8 years working in ED), ‘increased (infectious) disease, e.g., diarrhoea outbreaks secondary to both drought and low dam levels and floods’ (Participant 9, woman, 7 years working in ED) and ‘increase in respiratory illnesses from (air) pollution’ (Participant 5, woman, 4 years working in ED). Participants’ perceptions regarding the health impacts of climate change are contained in Table 2.

**TABLE 2:** Perceptions among emergency registrars and physicians regarding climate change’s health impacts.

Questions and responses	Frequency	%
**Do you think there is a link between climate change and health?**		
Definitely not	0	0
Probably not	0	0
Neutral	4	20
Probably	3	15
Definitely yes	13	65
**Please rate the extent to which you agree or disagree with the following statements: Climate change currently impacts population health outcomes**		
Strongly disagree	0	0
Disagree	0	0
Unsure	5	25
Agree	4	20
Strongly agree	11	55
**Climate change will affect the health of my future patients**		
Strongly disagree	0	0
Disagree	0	0
Unsure	1	5
Agree	6	30
Strongly agree	13	65
**Climate change will affect my personal health**		
Strongly disagree	1	5
Disagree	2	10
Unsure	3	15
Agree	7	35
Strongly agree	7	35
**Emergency care providers should know about climate change and its health impacts for clinical practice**		
Strongly disagree	0	0
Disagree	1	5
Unsure	2	10
Agree	5	25
Strongly agree	12	60

### Self-perceived preparedness to respond to the health impacts of climate change

Participants were asked to rate the educational climate resources that would be most useful to explore the interactions between climate change (or more broadly, the environment) and health, from a list of 13 options, including a free-text option. Participants chose the following five resources in order of importance: hospital system sustainability quality improvement projects (i.e., initiatives intended to improve the long-term feasibility and quality of in-hospital health care services), climate change advocacy (i.e., efforts to raise the awareness of climate change), accredited short courses, an elective course and events (e.g., expert panels and film screenings).

The participants’ perceived preparedness to respond to climate-related health impacts is contained in Table 3.

**TABLE 3:** Emergency medicine registrars’ and physicians’ perceived preparedness to respond to the health impacts of climate change.

Questions and responses	Frequency	%
**If a patient asked you, How can climate change impact my health? how prepared would you feel to engage in a conversation with them?**		
Not at all prepared	4	20
Very little prepared	5	25
Neither little nor much	8	40
Quite prepared	2	10
Very prepared	1	5
**Do you think that you have the information necessary to prepare for the health impacts of climate change (e.g., infectious disease and extreme weather events)?**		
Definitely not	1	5
Probably not	9	45
Neutral	7	35
Probably	3	15
Definitely yes	0	0
**How often do you receive training or workshops with regard to the health impacts of climate change in your line of duty?**		
Never	15	75
Seldom	4	20
Sometimes	1	5
Often	0	0
**Do you think you have received enough training or workshops in your line of duty with regard to climate change and health issues (e.g., impacts of climate change on infectious diseases)?**		
Definitely not	12	60
Probably not	6	30
Neutral	0	0
Probably	2	10
Definitely yes	0	0

### Perceptions, level of engagement and expectations related to climate change in medical education and training

All of the participants reported that they received no formal training regarding the health impacts of climate change during medical school. Participants were asked to provide their perceptions about climate-related health education at their medical school, and only 9 participants (representing 5 medical schools) responded to the question. Some participants described climate change as ‘a public health threat and priority’ (Participant 11, woman, 8 years working in ED) that ‘should be discussed and debated (e)specially at postgraduate/registrar level’ (Participant 9, woman, 7 years working in ED). One participant said: ‘Unless we start raising a generation of socially responsible professionals, we are doomed’ (Participant 19, woman, 26 years’ experience in the ED). Others highlighted the importance of including information about the regional effects of climate change, public health aspects, social determinants of health and how the political landscape impacts access to food and water. However, not everyone expressed a need for the integration of climate change in medical education and training. One participant wrote that while:

‘climate change is a major challenge … requiring mass cooperation and coordination … I do not find it pertinent to physicians in particular beyond being aware of the changes and how they impact our patients in reality (not how they might in future etc.).’ (Participant 16, man, 3 years working in ED)

The participants’ perceptions about climate-related health education are contained in Table 4.

**TABLE 4:** Emergency medicine registrars’ and physicians’ perceptions about climate change-related health education in health sciences curricula.

Questions and responses	Frequency	%
**Do you believe that the health impacts of climate change should be included in health sciences curricula?**		
Definitely not	0	0
Probably not	1	5
Neutral	2	10
Probably	10	50
Definitely yes	7	35
**At what levels do you think curriculum on climate change should be included in medical training?**		
Mandatory preclinical undergraduate medical curriculum	14	70
Postgraduate medical specialist training	8	40
Mandatory clinical rotation	4	20
Elective preclinical undergraduate medical curriculum	3	15
Elective clinical rotation	2	10
Postgraduate course	1	5
None of the above	1	5
**Do you think that you received enough formal training surrounding the health impacts of climate change?**		
Definitely not	7	35
Probably not	7	35
Neutral	5	25
Probably	1	5
Definitely yes	0	0
**How would you rate your level of engagement related to climate change during medical school?**		
Very high	0	0
High	0	0
Moderate	1	5
Low	15	75
None	4	20

### Perceived priorities to focus on that are expected to increase preparedness to respond to the health impacts of climate change

Participants were asked what, in their opinion, are the main priorities to focus on that will increase preparedness to respond to climate-related health impacts, and all 20 participants responded to this free-text question. The majority (65%, *n* = 13) of the participants emphasised the importance of ‘Education and information!’ (Participant 18, woman, 6 years working in ED). Participants emphasised a need for ‘increased awareness of climate change impact, prevention and preparation strategies’ (Participant 1, woman, 5 years working in ED), ‘knowledge on (the) effects of climate change on health’ (Participant 3, woman, 8 years working in ED) and ‘an awareness of epidemiological patterns of disease’ (Participant 16, man, 3 years working in ED). Other priorities included the identification of a network of experts ‘… to effectively bring climate advocacy into undergraduate medicine’ (Participant 19, woman, 26 years working in ED), a focus on the health impacts of climate change (e.g., infectious disease and food insecurity) as well as social determinants of health (e.g., poverty), and the availability of resources and ‘policies to deal with disaster management’ (Participant 8, woman, 7 years working in ED).

## Discussion

Most of the participants in this study recognised a link between climate change and population health and believed that climate change will impact their health and the health of their patients. This is in line with other studies across the globe that indicate that HCPs believe that climate change has or will harm human health.^8,10,29,30,31,32,33^ In a study conducted among 600 medical students from 12 universities across the US, 93.9% (*n* = 563) of the participants somewhat or strongly believed there is a link between climate change and population health, 90.8% (*n* = 545) believed that climate change will impact their personal health and 94.5% (*n* = 567) believed climate change will impact the health of their patients.^8^ It can therefore be said that many HCPs recognise the relationship between climate change and health.

However, the majority of the participants in this study reported feeling inadequately prepared to address climate-related health impacts in clinical practice and indicated that the training they had received was insufficient to prepare them for their current or future work as emergency specialists caring for patients in a changing climate. When asked what priorities should be addressed to improve their preparedness, participants identified education and information as key. Specifically, they called for a better understanding of the health impacts of climate change, including changing epidemiological patterns, infectious diseases and food insecurity, as well as better awareness of its intersections with social determinants of health, such as poverty, and policy guidance on disaster management.

It was therefore unsurprising that when asked whether the health impacts of climate change should be included in health sciences curricula, the majority of the participants in this study said they would probably or definitely support its inclusion. This gap in medical education, resulting in a lack of perceived preparedness to address climate-related health impacts, was echoed in several studies across the globe, including a multi-national survey conducted among HCPs from 12 health professional associations,^10^ and research from Germany,^9^ Tanzania,^34^ Ghana^12^ and Uganda.^35^ Considering that the link between climate change and health is a developing field,^17^ these results are not surprising, and medical students and educators worldwide have highlighted the need for training and education on the topic.^10,36,37,38,39^ As such, studies like the current study provide an essential departure point for bringing together health professions education and climate change.

Participants in this study reported that they did not receive any formal education or training about the health impacts of climate change during medical school. This is in line with other studies indicating low to no integration of climate-related health aspects in medical curricula,^11,13,40^ which highlights an ‘inconsistency between environmental changes, community health needs and medical education’^11, p. 1109^ and consequently a workforce that is not adequately prepared to discuss and address the health needs of the communities they serve.^11^ It can therefore be argued that a redesign of the medical curriculum, to include context-specific and relevant education to sufficiently prepare emergency medicine specialists to recognise and respond to the health impacts of a changing climate, is essential.^11,38^

However, it should be noted that while there appears to be a widespread call from HCPs, medical students and educators for the integration of climate-related health training and education into medical curricula,^10,29,31,32,33,36,37,38,39,41,42^ not all share this view. In a study conducted among medical students in the US, 4.3% (*n* = 26) of 600 respondents somewhat or strongly disagreed that physicians should know about the health impacts of climate change for clinical practice, while a further 4.2% (*n* = 25) neither agreed nor disagreed.^8^ The clinical relevance of teaching climate change was also questioned by one participant in this study. Other opposing perspectives point to structural barriers, such as an already crowded curriculum and a high burden of academic and clinical work.^10,30,33^ While such opposing views highlight important concerns that should be critically evaluated and addressed, they present an opportunity to re-evaluate how the interconnection between climate change and health is framed and presented. Instead of viewing the topic as an additional burden, the integration of climate-related health education in medical curricula could be seen as an opportunity to improve clinical practices^43^ and strengthen the understanding of the interplay between people and the environment and how this affects health^41^ while remaining practical and contextually relevant.

The question though remains about where in the medical curricula this topic should be introduced. In this study, participants mostly supported integration of the topic as a mandatory preclinical undergraduate level and as part of postgraduate medical specialist training. This is in line with a survey study conducted among medical students across the US, where participants most strongly supported integration of the topic either as a mandatory or elective at the preclinical level.^8^ In terms of preferred educational resources, aside from the more traditional didactic learning formats such as accredited short courses and elective modules, participants also prioritised hospital system sustainability quality improvement projects (i.e., initiatives designed to improve the long-term feasibility and quality of in-hospital health care services), climate change advocacy to raise awareness and events such as expert panels and film screenings. In line with recommendations that position HCWs as trusted and influential voices in the community that should build resilient and adaptive health care systems that protect individuals and communities against the health threats of climate change,^15,16^ these preferences reflect a need for practical, systems-oriented learning opportunities and a desire to actively engage in climate change advocacy.

Therefore, placing emergency medicine specialists at the face of the response to climate-related health impacts, which tends to disproportionately affect marginalised communities,^3^ then raises the question of whether a gap in medical education, potentially leading to an emergency workforce insufficiently prepared to address the health impacts of climate change, represents a climate justice issue that must not be overlooked.

Climate justice is crucial in the discussion about mitigative and adaptive actions to reduce climate-related impacts on communities in Africa^4^ as it views these impacts through a human rights lens, acknowledging the intersecting vulnerabilities and the disproportionate impact on marginalised groups.^3,4^ South Africa, which was recently declared the most unequal nation in the world,^5^ is no different in this regard. Climate impacts, such as health emergencies because of extreme weather events, compound existing social inequalities, intensifying poverty and widening the inequality gap.^2^ Pillai et al. suggest that:

‘as medical schools commit to training students to recognise injustice and promote health equity … these goals cannot be met without greater emphasis on environmental health – including climate science – in the curriculum.’^12, p1501^

We, therefore, argue that it is essential to include climate-related health education and training in the medical curriculum to sufficiently prepare emergency medicine specialists in South Africa to effectively care for those communities that will be most affected by climate change.

This aligns with Sustainable Development Goal (SDG) 13, which emphasises the integration of climate change-related education into curricula and policy to enhance awareness, knowledge and the capacity of HCWs in climate change adaptation, mitigation, early warning and impact reduction.^44^ A better understanding of the health impacts of climate change could enhance the involvement of emergency HCWs in implementing climate change mitigative and adaptive strategies^17^ and help mobilise a trusted and influential group of voices within communities to advocate for climate action.^18^ Future research should expand the sample to include other groups within emergency health care and explore the specific, context-appropriate learning objectives and goals.

### Strengths and limitations

The study made use of convenience sampling, which is susceptible to bias, and for that reason, the results of the study are not generalisable to the wider population. Despite efforts to encourage participation by allowing 3 months to respond to the survey and recirculating the invitation several times during this period, the response rate was low. While the exact response rate for this study is unknown because of the sampling technique that was used, it is estimated, based on the estimated population size of 200, that approximately 10% of the population responded to the survey. This is similar to other survey studies conducted in the field ranging between 10 and 23%.^8,10,14,45^ In a multinational survey study aimed at examining HCPs’ views of climate change as a health concern, the response rate varied from 0.4% to 22.5%, with the response rate in South Africa in that study being 3.2%.^10^ In another survey study exploring the perceptions of Faculty of Health Sciences students at the University of Cape Town on climate change and environmental sustainability, the response rate was 12.5%.^14^ Therefore, the response rate in this study seems to be in line with what was obtained in other similar studies.

To get an understanding of the extent of the bias, we compared the sample demographics to the population demographics. A retrospective review of the HPCSA’s database reported that there were 134 emergency medicine specialists registered (presumably in 2019), of which 83 (62%) participants were male and 51 (38%) participants were female.^46^ What this means is that the current sample was likely biased towards the female gender. This is not surprising, considering an implicit cognitive association between femininity and pro-environmental behaviour (PEB) (i.e., those who engage in PEB are perceived as more feminine and also perceive themselves as more feminine), and the tendency for women to be more concerned about climate change, which is partially explained by gender identity maintenance (i.e., conforming to gender expectations) and conversely threatens the gender identity of men (who are expected to not be feminine).^47^ While this study did not explore gender-based differences in perceptions or preparedness, future research could examine how gender may influence attitudes towards climate-related education, particularly in male-dominated specialities such as emergency medicine.

Furthermore, because of self-selection, it can be expected that those who have an interest in climate change may have been more likely to participate in the study, which could have resulted in an overestimation in the sample of the health impacts of climate change, the need to include the topic in medical curricula and perceived preparedness to deal with it when compared to the rest of the study population. However, as outlined in the discussion, the findings from this study resonate well with the findings from other studies across the globe.

Although demographic data were collected as part of this study, we did not analyse whether these characteristics (e.g., medical school attended and years of experience) influenced participants’ perceptions or self-reported preparedness. Given the exploratory and descriptive nature of the study and the small sample size, subgroup analyses were beyond the scope of the study. Nonetheless, we acknowledge that demographic factors may shape how emergency medicine specialists engage with climate-related health issues, and this warrants further investigation in future research with larger and representative samples.

Despite the limitations discussed above, this study has several methodological strengths. The use of an online survey design allowed us to collect data from participants who were geographically dispersed across South Africa within a relatively short period of time, facilitating broader representation than would have been feasible through in-person methods. In addition, by adapting items from two previously published studies conducted in different contexts, we were able to draw some quantitative comparisons across settings. Given that this is the first study to explore emergency medicine specialists’ perceptions and preparedness regarding the health impacts of climate change in South Africa, the survey approach provided a useful foundation for identifying key trends and generating questions for further research.

## Conclusion

Emergency health care is often at the forefront of the response to climate-related health impacts, and therefore, it plays an essential role in the health care system, driving adaptive strategies to better prepare for these challenges. However, to date, in the South African context, little is known about emergency HCWs’ perceptions of and preparedness to respond to the health impacts of climate change. This study aimed to provide an initial description of this cadre’s perceptions of climate change and health, climate-related health education and their preparedness to respond.

The results indicated that most participants recognised a relationship between climate change and health and believed it would impact both their own and their patients’ health. However, they reported having received no formal training during medical school, and the majority felt inadequately prepared to respond to these health impacts in clinical practice. It was therefore unsurprising that most participants highlighted the need to integrate aspects of the topic into medical curricula to adequately prepare them to respond to these impacts in clinical practice. These findings suggest a gap in preparedness among emergency HCWs in South Africa and underscore the need for a climate-responsive emergency care curriculum.

To our knowledge, this is the first study exploring emergency medicine registrars’ and physicians’ perceptions and preparedness as it relates to the health impacts of climate change and climate change in medical education. These findings highlight an emerging awareness of the relevance of climate change to clinical practice and a perceived gap in preparedness and formal training. As such, the results may inform future research aimed at defining specific educational needs of emergency medicine specialists and guiding the early development of climate-health educational initiatives, including accredited short courses, elective modules, practical, systems-oriented learning opportunities and opportunities for climate change advocacy.
